# The association of copeptin with metabolic risk markers is modified by region of origin

**DOI:** 10.1038/s41598-023-46908-0

**Published:** 2023-11-10

**Authors:** Anna Franzén, Miriam Pikkemaat, Olle Melander, Louise Bennet, Sofia Enhörning

**Affiliations:** 1 Department of Clinical Sciences in Malmö, Lund University, Clinical Research Center 91:12, Jan Waldenströms gata 35, 21428 Malmö, Sweden; 2 Department of Internal Medicine, Skåne University Hospital, Malmö, Sweden; 3 Clinical Studies Sweden, Forum South, Skåne University Hospital, Lund, Sweden

**Keywords:** Endocrine system and metabolic diseases, Risk factors

## Abstract

Iraqi born immigrants in Sweden have higher prevalence of metabolic diseases compared to native Swedes. Copeptin, a marker for vasopressin, is associated with increased risk of metabolic disease. In this cross-sectional population study based on the MEDIM cohort we investigated differences in copeptin levels between Iraqi and Swedish born individuals and if the association between copeptin and cardiometabolic risk markers differed by region of origin. We included 1109 Iraqi and 613 Swedish born participants (58% men, mean age 47 years). The Swedish participants had a higher concentration of copeptin compared to the Iraqi born group after age and sex adjustment (*p* < 0.001). This difference existed only among male individuals with the highest copeptin concentrations, i.e. belonging to copeptin quartile 4 (median (25th; 75th percentile) 20.07 (15.27;33.28) pmol/L for the Swedish born versus 15.57 (13.91;19.00) pmol/L for the Iraqi born, *p* < 0.001). We found a significant interaction between copeptin (continuous ln-transformed) and being born in Iraq regarding the association with plasma triglycerides (*P*_interaction_ = 0.006). The association between copeptin and BMI was stronger amongst the Iraqi born individuals compared to the Swedish born. Together, this could indicate that copeptin is a more potent marker of metabolic disease among individuals born in Iraq compared to Sweden.

## Introduction

The impact of metabolic risk factors on health has almost doubled over the last decade. Today the metabolic risk factors constitute the greatest of all preventable risk factors of disease, according to The Global Burden of Disease. Together, high blood pressure, blood sugar, body mass index and blood cholesterol were responsible for almost 27 million deaths in 2019. The risk factors are major contributors to development of diabetes and cardiovascular disease (CVD)^1^.

In Sweden immigrants from Iraq, who constitutes one of the larger immigrant groups, have twice the prevalence of type 2 diabetes (T2D) compared to Swedish born individuals according to the MEDIM study (Impact of Migration and Ethnicity on Diabetes in Malmö)^2^. The metabolic profile in general is more disadvantageous among Iraqi born individuals, with higher levels of plasma triglycerides (p-TG)^3^, higher prevalence of obesity and higher fatty liver index^4^.

The hormone vasopressin (VP) has raised more attention lately, due to its involvement in the development of metabolic disease. VP can be called antidiuretic hormone and is released from the pituitary gland primarily in response to high plasma osmolality^5^. The hormone is also involved in the glucose and fat metabolism stimulating glucogenesis and glycogenolysis and synthesis of triglycerides in the liver^6^, glucagon secretion from the pancreas^7^, adrenocorticotropic hormone (ACTH) release from the pituitary gland^8^ and cortisol secretion from the adrenal gland^9^. Since VP is known to be unstable, rapidly cleared from plasma and not easily measured, it is disadvantageous to use as a biomarker. Copeptin, the c-terminal fragment of the VP precursor prepro-VP, is released in equimolar quantities to VP and is a reliable marker of the VP secretion^10^. Copeptin is previously associated with metabolic diseases including T2D^11^, overweight^6^, the metabolic syndrome^12^ and fatty liver^13^. High copeptin levels are also independently associated with increased risk of kidney disease^14^ and cardiovascular events^15^.

The exact mechanisms behind the differences in risk of metabolic disease between Swedish born and Iraqi born individuals are not completely clear and can not be fully explained by differences in traditional life-style factors^2^. Additionally, previous studies have pointed out that copeptin levels may differ by ethnicity^15,16^, even though contradictory results also exists^13^. Since VP is causally linked to metabolic disease, we hypothesized in this study that ethnical differences in VP concentration may provide one possible explanation behind the different risk profile.

The aims of this study were two-fold. First, we wanted to investigate if copeptin concentration differed between Iraqi born and Swedish born individuals in the MEDIM cohort, two groups living in the same area, with known differences in prevalence and incidence of metabolic diseases. Second, we wanted to investigate if copeptin was associated with markers of cardiometabolic disease and if these associations differed between region of origin.

## Results

### Description of the study population

**Figure 1:**
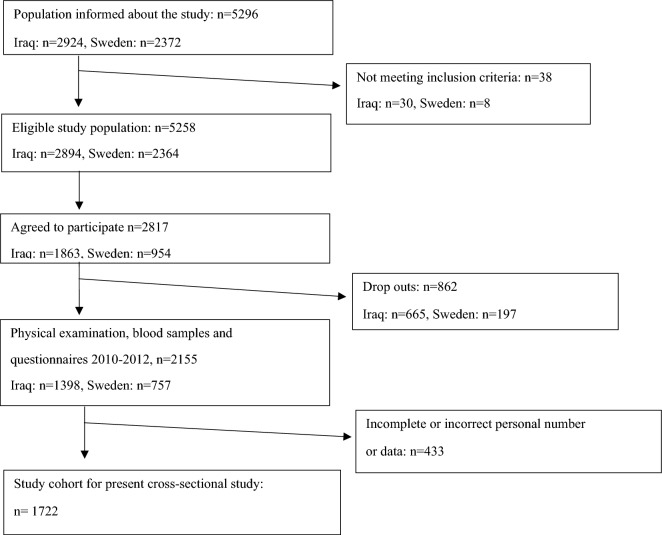
Flow diagram of study population.

Out of the 1398 Iraqi-born and 757 Swedish born individuals, 289 Iraqi born, and 144 Swedish-born individuals were excluded from the analysis due to incomplete or missing data, resulting in 1109 Iraqi born and 613 Swedish born individuals included in the further analysis of the current study (Fig. 1). Baseline characteristics are presented in Table 1. The Iraqi born participants were younger and had a higher proportion of male participants. They were less physically active, had a higher body mass index (BMI) and a larger waist circumference, had a higher prevalence of T2D and a larger proportion of participants had economic difficulties. The Swedish born population had a higher prevalence of hypertension and a higher intake of alcohol.

**Table 1:** Study population description (n = 1722).

	Born in Sweden (n = 613)	Born in Iraq (n = 1109)	*P* value^2^
Age in years	49.5 (11.4)	45.9 (9.4)	** < 0.001**
Sex (proportion of men, n (%))	329 (53.7%)	664 (59.9%)	**0.01**
p-copeptin (ln-transformed)	2.02 (0.83)	1.94 (0.61)	**0.04**
p-copeptin^1^ (pmol/L)	6.64 (4.41;10.52)	6.77 (4.46;10.29)	0.86
p-glucose (mmol/L)	5.72 (0.98)	5.92 (1.49)	** < 0.001**
p-triglycerides (ln-transformed)	0.07 (0.52)	0.31 (0.55)	** < 0.001**
p-triglycerides^1^ (mmol/L)	1.00 (0.80;1.50)	1.40 (0.90;2.00)	** < 0.001**
High density lipoprotein (mmol/L)	1.44 (0.44)	1.20 (0.34)	** < 0.001**
Low density lipoprotein (mmol/L)	3.29 (0.88)	3.19 (0.84)	**0.02**
Body mass index (kg/cm^2^)	27.2 (4.6)	29.1 (4.3)	** < 0.001**
Waist circumference (cm)	93.53 (13.27)	96.43 (10.96)	** < 0.001**
Systolic blood pressure (mmHg)	135.6 (20.0)	128.8 (16.6)	** < 0.001**
Diastolic blood pressure (mmHg)	80.9 (11.6)	78.0 (10.4)	**0.01**
Estimated glomerular filtration (ml/min)	93.4 (14.9)	98.7 (15.6)	** < 0.001**
Alcohol intake (standard glasses per week)	1.81 (1.25)	0.37 (0.88)	** < 0.001**
Prevalent diabetes, n (%)	37 (6.0%)	111 (10.0%)	**0.005**
Prevalent hypertension, n (%)	264 (43.1%)	356 (32.1%)	** < 0.001**
Physical activity < 30 min/day	225 (36.7%)	786 (70.9%)	** < 0.001**
Current smokers, n (%)	153 (25.0%)	268 (24.2%)	0.73
Economic difficulties, n (%)	54 (8.8%)	400 (36.1%)	** < 0.001**

Bold numbers indicate significant effects.

Values are presented as mean (s.d.) if not otherwise specified.

^1^Expressed as median (25th; 75th) percentile.

^2^*P* value based on t-test when comparing means, Mann–Whitney U-test when comparing medians and Chi-Square test when comparing categorical variables.

### Copeptin concentration

The Swedish born population had a slightly higher concentration of ln-transformed copeptin compared to the Iraqi born population (Table 1), This difference was still evident after adjustment for age and sex (*p* < 0.001) and remained significant after further adjustment for cardiometabolic risk markers that differed between country of birth in Table 1 (*p* < 0.001), i.e. high density lipoprotein (HDL), low density lipoprotein(LDL), p-TG, BMI, waist circumference, prevalent diabetes, prevalent hypertension, alcohol intake, physical activity and socioeconomic status. When we analysed differences in copeptin concentration stratified by sex in each copeptin quartile separately, the difference in copeptin concentration between Iraqi born and Swedish born individuals remained significant among men in quartile 4 solely (Table 2).

**Table 2:** Copeptin concentration per quartile of copeptin in Swedish born and Iraqi born individuals respectively.

		Born in Sweden, n = 613		Born in Iraq, n = 1109		*P* value^4^
All						
Quartile 1^1^	Median^2^	3.36 (2.85;4.26)	N = 158	3.48 (2.92;4.60)	N = 272	0.15
	Ln copeptin^3^	1.23 (0.29)		1.27 (0.30)		
Quartile 2^1^	Median^2^	5.89 (4.47;6.96)	N = 136	6.12 (4.40;7.33)	N = 294	0.45
	Ln copeptin^3^	1.73 (0.25)		1.75 (0.27)		
Quartile 3^1^	Median^2^	8.59 (6.06;10.30)	N = 147	8.96 (6.20;10.50)	N = 285	0.15
	Ln copeptin^3^	2.07 (0.28)		2.11 (0.28)		
Quartile 4^1^	Median^2^	15.27 (9.48;21.73)	N = 172	13.84 (10.43;16.77)	N = 258	** < 0.001**
	Ln copeptin^3^	2.93 (0.90)		2.67 (0.52)		
Women						
Quartile 1	Median^2^	2.99 (2.57;3.33)	N = 76	2.93 (2.60;3.26)	N = 106	0.67
	Ln copeptin^3^	1.04 (0.20)		1.03 (0.19)		
Quartile 2	Median^2^	4.41 (4.04;4.77)	N = 60	4.30 (3.97;4.68)	N = 122	0.18
	Ln copeptin^3^	1.48 (0.09)		1.46 (0.10)		
Quartile 3	Median^2^	6.05 (5.53;6.64)	N = 71	5.97 (5.58;6.49)	N = 112	0.66
	Ln copeptin^3^	1.80 (0.11)		1.80 (0.10)		
Quartile 4	Median^2^	9.11 (8.24;12.85)	N = 77	9.62 (8.33;11.55)	N = 105	0.20
	Ln copeptin^3^	2.45 (0.66)		2.34 (0.36)		
Men						
Quartile 1	Median^2^	4.20 (3.63;4.88)	N = 82	4.36 (3.56;5.09)	N = 166	0.46
	Ln-copeptin^3^	1.40 (0.25)		1.42 (0.25)		
Quartile 2	Median^2^	6.79 (6.13;7.77)	N = 76	7.16 (6.51;7.78)	N = 172	0.06
	Ln-copeptin^3^	1.93 (0.12)		1.96 (0.11)		
Quartile 3	Median^2^	10.14 (9.41;11.11)	N = 76	10.16 (9.29;11.17)	N = 173	0.70
	Ln-copeptin^3^	2.33 (0.11)		2.32 (0.11)		
Quartile 4	Median^2^	20.07 (15.27;33.28)	N = 95	15.57 (13.91;19.00)	N = 153	** < 0.001**
	Ln-copeptin^3^	3.32 (0.89)		2.89 (0.50)		

Bold numbers indicate significant effects.

^1^Sex-specific quartiles.

^2^Expressed as median (25th;75th) percentile (pmol/L).

^3^Expressed as mean ln-transformed copeptin (standard deviation).

^4^*P* value from t-test on ln-transformed copeptin.

### Descriptive statistic of men belonging to the quartile with the highest copeptin levels

To further investigate the traits of the individuals driving the difference in copeptin concentration between Iraqi and Swedish born participants, we studied men within copeptin quartile 4 in further detail (Supplemental Table 1). We found that, as in the entire population, the Iraqi born men had a higher BMI and larger waist circumference. Further, and in consistency with the entire population, they were less physically active and had a higher prevalence of T2D and to a higher extent economic difficulties, whereas the Swedish born men had a higher intake of alcohol.

### Association between copeptin levels and cardiometabolic risk markers

After multivariate adjustment we found significant associations between copeptin and increased glucose concentration, p-TG and BMI, respectively, and between copeptin and decreased estimated glomerular filtration rate (eGFR) (Table 3). These associations were significant after adjustment for current medication (antihypertensive medication, hypolipidemic medication and diabetes medication) on top of age, sex, and country of birth (*p* < 0.05 for all).

**Table 3:** Association between copeptin levels and cardiometabolic risk markers.

Dependent variable	Beta	95% CI	*P* value model 1	*P* value model 2
Glucose (mmol/L)	0.11	0.01;0.20	**0.03**	**0.01**
High density lipoprotein (mmol/L)	− 0.01	− 0.03;0.02	0.68	0.87
Low density lipoprotein (mmol/L)	− 0.01	− 0.08;0.05	0.65	0.43
Triglycerides^1^ (mmol/L)	0.03	0.01;0.05	** < 0.001**	**0.004**
Body mass index (kg/cm^3^)	0.40	0.06;0.70	**0.02**	**0.04**^**2**^
Waist circumference (cm)	0.68	− 0.13;1.48	0.10	0.55
Systolic blood pressure (mmHg)	1.13	− 0.01;2.31	0.06	0.31^3^
Diastolic blood pressure (mmHg)	0.31	− 0.44;1.06	0.41	0.89^3^
Estimated glomerular filtration rate (ml/min)	− 2.47	− 3.35; − 1.57	** < 0.001**	** < 0.001**

Data based on multivariate linear regression models, expressed as unit change in dependent variable per unit increase in continuous ln-copeptin.

Bold number indicate significant effects.

Model 1: Adjusted for age, sex and country of birth.

Model 2: Adjusted for age, sex, country of birth, socioeconomics, physical activity, smoking, alcohol intake, body mass index, diabetes and hypertension.

^1^Given as ln-transformed value.

^2^Not adjusted for body mass index.

^3^Adjusted for antihypertensive medication. Not adjusted for hypertension.

### Interaction between copeptin, country of birth and cardiometabolic risk markers

We investigated whether there were any interactions between copeptin and country of birth (copeptin*country of birth) regarding the association with cardiometabolic risk markers. Using linear regression with the interaction term as the independent variable and the cardiometabolic risk markers as the dependent variable, we found a significant interaction between continuous copeptin and Iraqi ethnicity on the association with increased p-TG (P_interaction_ = 0.006). We found no significant interaction between copeptin and ethnicity when investigating the other cardiometabolic risk markers that were significantly associated with copeptin in Table 3 (i.e. glucose, BMI and eGFR).

### The association between copeptin and cardiometabolic risk markers stratified according to country of birth

Finally,﻿﻿ ﻿﻿﻿we investigated whether copeptin was associated with metabolic risk markers in analyses stratified by country of birth (Table 4). In the Iraqi-born group, we found in multivariate adjusted models that increasing copeptin was significantly associated with increased fasting plasma glucose (fp-glucose), p-TG and BMI and with decreased eGFR. In the Swedish born group, there was a significant association between increased copeptin and increased fp-glucose and decreased eGFR, respectively.

**Table 4:** Association between copeptin levels and cardiometabolic risk markers in data stratified according to country of birth.

	Born in Sweden				Born in Iraq			
Dependent variable	Beta	95% CI	*P* value model 1	*P* value model 2	Beta	95% CI	*P* value model 1	*P* value model 2
Glucose (mmol/L)	0.11	0.01;0.20	**0.03**	**0.02**	0.09	− 0.06;0.24	0.25	**0.04**
High density lipoprotein (mmol/L)	0.01	− 0.03;0.05	0.58	0.38	− 0.02	− 0.06;0.01	0.21	0.36
Low density lipoprotein (mmol/L)	− 0.08	− 0.17;0.01	0.07	0.053	0.06	− 0.03;0.15	0.17	0.31
Triglycerides^1^ (mmol/L)	0.01	− 0.02;0.03	0.55	0.66	0.05	0.03;0.08	** < 0.001**	** < 0.001**
Body mass index (kg/cm^3^)	0.15	− 0.31;0.62	0.52	0.67^2^	0.61	0.16;1.06	**0.01**	**0.02**^**2**^
Waist circumference (cm)	0.03	− 1.23;1.39	0.96	0.48	1.32	0.25;2.39	**0.02**	0.69
Systolic blood pressure (mmHg)	0.70	− 1.12;2.51	0.45	0.61^3^	1.69	0.10;2.68	**0.04**	0.16^3^
Diastolic blood pressure (mmHg)	0.03	− 1.03;1.15	0.83	1.00^3^	0.67	− 0.36;1.70	0.20	0.47^3^
Estimated glomerular filtration rate (ml/min)	− 2.25	− 3.45; − 1.05	** < 0.001**	** < 0.001**	− 2.70	− 3.99; − 1.40	** < 0.001**	** < 0.001**

Data based on multivariate linear regression models, expressed as unit change in dependent variable per unit increase in continuous ln-copeptin.

Bold numbers indicate significant effects.

Model 1: Adjusted for age, sex and country of birth.

Model 2: Adjusted for age, sex, country of birth, socioeconomics, physical activity, smoking, alcohol intake, body mass index, diabetes and hypertension.

^1^Given as ln-transformed value.

^2^Not adjusted for body mass index.

^3^Adjusted for antihypertensive medication. Not adjusted for hypertension.

## Discussion

The most important findings in this study was that Swedish born individuals had significantly higher levels of plasma copeptin as compared to the Iraqi immigrants, and that this difference was only evident among men with the highest copeptin levels, i.e. belonging to copeptin quartile 4. This was unexpected, since copeptin is known to be associated with metabolic disease which is more prevalent among Iraqi born individuals. On the other hand, the links between copeptin and cardiometabolic risk markers seemed to be more pronounced among Iraqi born individuals, confirmed by an interaction between country of birth and copeptin on the associations with plasma-TG.

In consistency with previous studies^12,17^ we found associations between copeptin and several of the cardiometabolic risk markers in the complete cohort (p-glucose, p-TG, BMI, and eGFR). These results were also supported by previous studies showing elevated copeptin in several cardiometabolic conditions such as the metabolic syndrome, hypertension, and microalbuminuria^12,18^.

As there was a significant interaction between copeptin and Iraqi ethnicity on the association with increased p-TG, we chose to analyse the associations between copeptin and risk markers of cardiometabolic disease in Swedish and Iraqi born individuals separately. We found that the relationship between copeptin and markers linked to fat metabolism, i.e. TG and BMI, was only evident in the Iraqi born part of the population. It is previously known that Iraqi born immigrants have a higher prevalence of obesity and higher TG^19,20^. Based on the results of the current study, it would be interesting to further investigate if copeptin contributes to the more disadvantageous metabolic profile seen in the Iraqi born part of the population.

The VP hormone exerts its effects on metabolism in several ways by receptors in the liver, pancreas, and anterior pituitary gland^7–9^. In this study, the results indicate that the association between high copeptin levels and an unfavourable metabolic profile are more pronounced in individuals born in Iraq compared to Swedish born individuals. Previous data suggest a casual effect of VP on metabolic health^11,21–23^. Even though there are few studies investigating ethnical differences in copeptin, there are previous data indicating that the applicability of copeptin as a biomarker may differ between ethnical groups^16^. Therefore, one may speculate that the ability of copeptin to predict metabolic disease might differ between Middle Eastern and Swedish ethnicity, for example due to differences in expression or function of VP-receptors, making individuals from Iraq more susceptible to VP-exposure. This should be studied further in longitudinal settings as well as in studies investigating differences between ethnicity regarding genetic variation and receptor expression.

The significant difference in copeptin levels between the Iraqi and Swedish born individuals was driven by the Swedish born men with the highest copeptin. Previous studies have shown that copeptin can be elevated in response to various diseases, including cardiovascular and metabolic diseases, infectious diseases and vasodilatory shock^12,24–26^. Swedish born men belonging to copeptin quartile 4 had a higher alcohol intake than their Iraqi counterpart. Otherwise, we did not discover any differences regarding cardiometabolic risk markers in men belonging to quartile 4 that could possibly explain the significantly higher copeptin concentration among Swedish born men. Oppositely, we found slightly higher BMI and waist circumference and lower HDL among the Iraqi born men of quartile 4, which are all phenotypic traits that are linked to elevated copeptin concentrations. In healthy individuals, we previously found that elevated copeptin concentration was linked to relative underhydration^17,27^. This is not surprising, since VP is the key hormone regulating water balance in the body, and is, under physiological circumstances, mainly secreted as a response of increased plasma osmolality^5,28^. In addition, relative underhydration is rather common in the population^29,30^. Thus, one may speculate that the elevated copeptin levels among Swedish born men compared to Iraqi born men found in this study may be due to insufficient fluid intake, perhaps in combination with higher alcohol induced diuresis^31^. In this context it is worth to mention that alcohol is commonly said to inhibit the release of VP independently of plasma osmolality, but experiments have failed to prove this hypothesis^31,32^. In this study we do not have any data on urine output, fluid intake or other markers of hydration, restraining us from investigating the hypothesis of underlying underhydration as a mechanism of elevated copeptin further.

As far as we know, this is the first study to investigate differences in copeptin concentration in a Middle-Eastern and Swedish born population. The potential of copeptin as a risk marker and prognostic marker of cardiometabolic disease have been shown repeatedly in different studies. To be able to use copeptin in a clinical setting it needs to be investigated and evaluated in different populations and ethnical groups. Our study confirms that copeptin is associated with metabolic risk markers also in Swedish individuals originating from the Middle-East.

The study is limited by its cross-sectional design which makes it impossible to draw any conclusions about causality. To investigate whether high copeptin predicts the risk of metabolic disease differently in the Iraqi born compared to the Swedish born population, longitudinal studies should be performed. Another limitation is that we do not have any data on fluid intake or other markers of hydration status in this material, why we can only speculate in the possible effects of underhydration as a reason behind elevated copeptin. The statistical analyses might be underpowered, especially when we stratified the analyses into Iraqi born and Swedish born individuals.

In this study, we conclude that compared to the Iraqi born, the Swedish born participants had a higher copeptin concentration, an association that was driven by the Swedish born men with the highest copeptin concentrations. Nevertheless, the relationship between elevated plasma copeptin and markers of fat metabolism seemed to be more pronounced among the Iraqi born individuals. Furthermore, we found a significant interaction between copeptin and Iraq as the country of birth on the association with elevated p-TG. Taken together, this could possibly indicate that copeptin is a more potent marker of metabolic disease in the Iraqi born group as compared to the Swedish born group.

## Methods

### Study population

The study was based on the cohort in the MEDIM study. The baseline investigation in the MEDIM study was conducted between the years of 2010 and 2012 in Malmö, Sweden. Using the census register, Iraqi born immigrants between the age of 30 and 75 were invited. Swedish born citizens were thereafter randomly selected from the same area in Malmö to match the Iraqi born in gender and age distribution. Individuals were contacted by mail and phone and invited to the study. Individuals with type 1 diabetes mellitus, severe physical or mental illness or disabilities were excluded. 1398 Iraqi born immigrants and 757 Swedish born individuals were included in the study.

### Laboratory methods

Copeptin was analyzed in biobanked plasma samples from the MEDIM baseline investigation by using a KRYPTOR Compact Plus device and a commercially available chemiluminescence sandwich immunoassay copeptin proAVP kit with coated tubes from samples stored at − 80 °C (BRAHMS Copeptin proAVP KRYPTOR; ThermoFisher Scientific). All other laboratory analyses were performed at the time of the MEDIM baseline investigation and are explained in detail previously^33^.

### Study variables

All participants were physically examined during the MEDIM baseline study in the year of 2010–2012. The examination was performed by trained research nurses and included height, weight, waist circumference and blood pressure. Blood pressure was measured in a supine position after 5 min rest. In connection to this, nurses used a structured form to collect information about current medication, diabetes diagnosis, physical activity, and socioeconomics. All participants not previously diagnosed with diabetes, conducted an oral glucose tolerance test (OGTT). Diabetes prevalence in this study was defined as a fasting glucose level ≥ 7.0 mmol/L and/or an oral glucose tolerance test (OGTT) ≥ 11.1 mmol/L after 2 h. If only one of these two values exceeded the thresholds, OGTT was performed again another day within two weeks as two pathological values were needed to obtain diabetes diagnosis. Hypertension was defined as follows: systolic blood pressure ≥ 140 mmHg, diastolic blood pressure ≥ 90 mmHg or use of antihypertensive medication. The socioeconomic variable was based on self-reported data of economic difficulties defined as difficulties to pay rent or bills on more than one occasion for the last 12 months. The physical activity variable was based on self-reported data and physical inactivity was defined as < 30 min a day spent on non-strenuous or strenuous activities. Data on smoking is based on self-reported smoking the last 6 months. Data on alcohol intake is based on self-reported estimation of the number of standard glasses consumed per week.

### Statistics

The statistical analyses were performed in SPSS version 27 and 29 (IBM cooperation ®).

Continuous variables were presented as means with standard deviation, categorical variables as frequencies and percentages. Non-normally distributed data (i.e. copeptin and TG) were presented as medians with interquartile range. T-test was performed to analyse differences between Iraqi born and Swedish born citizens for continuous normally distributed variables, Mann–Whitney U-test was used for non-normally distributed variables and Chi-square test was used for categorical variables. Ln-transformed values were used for copeptin and TG when used in the linear regression models. Normality was tested by plotting data graphically in a histogram.

Using multivariate linear regression models, we investigated whether copeptin was associated with cardiometabolic risk markers after adjustment for age, sex, and country of birth in a first model and with additional adjustment for socioeconomics, BMI (except for when BMI was the outcome variable), physical activity, alcohol consumption, smoking, diabetes and hypertension (except for when systolic blood pressure (SBP) and diastolic blood pressure (DBP) was the outcome variable) in a second model. Non-normally distributed variables (i.e. copeptin and TG) were ln-transformed before analysis. Subsequently, an interaction term between continuous ln-transformed copeptin and region of origin (copeptin*country of birth) was introduced in the sex-adjusted linear regression model to analyse the association with markers of metabolic disease. When analysing differences between ethnicity in each copeptin quartile, sex-specific quartiles of copeptin concentration were used. The results were considered statistically significant when the two-sided *p* value was < 0.05.

### Ethics declarations

The study was performed in line with standards declared in the 1964 Declaration of Helsinki and its later amendments and other relevant guidelines. All of the study participants have given an informed and written consent. The Swedish Ethical Review Authority approved the MEDIM study (Dnr 2009/36, and 2019/01166).

### Supplementary Information

Supplementary Information.

## Data Availability

The dataset analysed during the current study can be provided from the corresponding author on reasonable request.

## Code availability

The codes used for the statistical analyses can be provided upon request from the corresponding author.
