# Supplementary material for: The association of copeptin with metabolic risk markers is modified by region of origin
Source: Sci Rep. 2023 Nov 10;13:19651. doi: 10.1038/s41598-023-46908-0 (PMC10638355; doi:10.1038/s41598-023-46908-0)
Supplement: Supplementary file 1 — Supplementary Information. [file 41598_2023_46908_MOESM1_ESM.docx]

**Supplements**

**Table 1. Descriptive characteristics of men belonging to copeptin quartile 4**

|  | **Born in Sweden (n=95)** | **Born in Iraq (n=153)** | **p-value** |
| --- | --- | --- | --- |
| Age in years | 45.58 (11.58) | 47.18 (9.99) | 0.79 |
| Copeptin (ln-transformed) | 3.32 (0.89) | 2.89 (0.50) | **<0.001** |
| Copeptin (pmol/L)^1^ | 20.07 (15.27;33.28) | 15.57 (13.91;19.00) | **<0.001** |
| p-glucose (mmol/L) | 5.83 (1.10) | 6.01 (1.79) | 0.34 |
| Triglycerides (ln-transformed) | 0.10 (0.24) | 0.23 (0.25) | **<0.001** |
| p-triglycerides^1^ (mmol/L) | 1.20 (0.90;1.90) | 1.70 (1.10;2.50) | **<0.001** |
| High density lipoprotein (mmol/L) | 1.28 (0.38) | 1.09 (0.29) | **<0.001** |
| Low density lipoprotein (mmol/L) | 3.31 (0.89) | 3.33 (0.86) | 0.89 |
| Body mass index (kg/cm^2^) | 27.16 (4.14) | 29.45 (4.27) | **<0.001** |
| Waist circumference (cm) | 97.21 (12.23) | 100.50 (11.41) | **0.03** |
| Systolic blood pressure (mmHg) | 136.29 (16.80) | 132.83 (17.44) | 0.12 |
| Diastolic blood pressure (mmHg) | 80.95 (10.01) | 80.24 (11.14) | 0.61 |
| Estimated glomerular filtration rate (ml/min) | 92.31 (14.51) | 93.30 (16.34) | 0.63 |
| Prevalent diabetes, n (%) | 6 (6.3%) | 18 (11.8%) | 0.19 |
| Prevalent hypertension, n (%) | 42 (44.2%) | 63 (41.2%) | 0.69 |
| Physical activity <30 min/day | 38 (40.0%) | 103 (67.3%) | **<0.001** |
| Current smokers, n (%) | 19 (20.0%) | 47 (30.7%) | 0.08 |
| Economic difficulties, n (%) | 5 (5.3%) | 58 (37.9%) | **<0.001** |
| Alcohol intake (standardglasses per week) | 2.13 (1.31) | 0.60 (1.09) | **<0.001** |
| Bold numbers indicate significant effects. Values are presented as mean (s.d.) if not otherwise specified.  P-value based on t-test when comparing means, Mann-Whitney U-test when comparing medians and Chi-Square test when comparing categorical variables.  ^1^ Expressed as median (25^th^;75^th^) percentile. Copeptin concentrations (median (min-max) pmol/L) within copeptin quartile 4 were among Swedish born men 20.07 (12.47-491.30) for and among Iraqi born men 15.57 (12.41-356.20). | | | |
